# Nuplazid suppresses esophageal squamous cell carcinoma growth in vitro and in vivo by targeting PAK4

**DOI:** 10.1038/s41416-021-01651-z

**Published:** 2021-12-15

**Authors:** Yaxing Wei, Wenjie Wu, Yanan Jiang, Hao Zhou, Yin Yu, Lili Zhao, Xiangyu Wu, Xuebo Lu, Qiang Yuan, Zitong Wang, Zigang Dong, Luyun He, Jimin Zhao, Kangdong Liu

**Affiliations:** 1 Pathophysiology Department, School of Basic Medical Sciences, Zhengzhou University, Zhengzhou, Henan China; 2 China-US (Henan) Hormel Cancer Institute, Zhengzhou, Henan China; 3 Provincial Cooperative Innovation Center for Cancer Chemoprevention, Zhengzhou University, Zhengzhou, Henan China; 4 State Key Laboratory of Esophageal Cancer Prevention and Treatment, Zhengzhou, Henan China; 5 Cancer Chemoprevention International Collaboration Laboratory, Zhengzhou, Henan China

**Keywords:** Cancer prevention, Cancer prevention

## Abstract

**Background:**

Due to the high recurrence and low 5-year survival rates of esophageal squamous cell carcinoma (ESCC) after treatment, the discovery of novel drugs for recurrence chemoprevention is of particular importance.

**Methods:**

We screened the FDA-approved drug library and found that Nuplazid, an atypical antipsychotic that acts as an effective 5-HT 2 A receptor inverse agonist, could potentially exert anticancer effects in vitro and in vivo on ESCC.

**Results:**

Pull-down results indicated that Nuplazid binds with p21-activated kinase 4 (PAK4), and a kinase assay showed that Nuplazid strongly suppressed PAK4 kinase activity. Moreover, Nuplazid exhibited inhibitory effects on ESCC in vivo.

**Conclusions:**

Our findings indicate that Nuplazid can suppress ESCC progression through targeting PAK4.

## Background

Esophageal cancer is the seventh most commonly diagnosed cancer and the sixth most common cause of cancer-related mortality worldwide [1]. Esophageal squamous cell carcinoma (ESCC) accounts for ~90% of esophageal cancers [2], and is typically treated with surgery, chemotherapy, radiotherapy and combination therapy [3, 4]. However, even with treatment, the 5-year overall survival rate has been reported to be ~47%, with 49% of patients developing locoregional progression or distant progression [5, 6]. Therefore, it is important to identify novel drugs with low toxicity and high efficacy to prevent the recurrence of ESCC and increase the survival rate of patients. The repositioning of drugs can overcome the challenges of high wastage, high cost, and time-consuming drug development [7, 8]. Screening drugs approved by the FDA and repositioning their anti-tumour effects have the potential to overcome several challenges associated with drug development and to guarantee rapid clinical trials.

p21-Activated kinase 4 (PAK4) is involved in numerous signaling pathways and plays a pivotal role in cytoskeleton regulation, cell migration, growth, proliferation and survival [9]. The overexpression of PAK4 is reported to be closely related to the occurrence and development of various cancers, including pancreatic [10], breast [11, 12], ovarian [13], and gastric cancers [14]. Currently, several compounds have been identified as PAK4 inhibitors, which typically target the ATP-binding pocket of PAK4 kinase domain [15]. However, the transition from compound inhibitors to clinical medications needs to overcome time-consuming and exorbitant cost factors.

Nuplazid, an atypical antipsychotic that functions as an effective 5-HT 2 A receptor inverse agonist, is mainly used to treat Parkinson’s disease psychosis [16]. In this study, we screened drugs approved by the FDA and found Nuplazid could inhibit the growth of ESCC in vitro. Then our study found that Nuplazid treatment inhibits the growth of ESCC by binding to PAK4 and regulating its downstream signaling pathway interaction. The anticancer effects of Nuplazid on ESCC in vitro and in vivo suggested Nuplazid might be a candidate for ESCC chemoprevention.

## Methods

### Cell culture

The Shantou human embryonic esophageal (SHEE) cell line was obtained from Dr. Enmin Li (Medical College of Shantou University) [17]. Human esophageal cancer cell lines KYSE150, KYSE410 and KYSE450 cells were purchased from the Type Culture Collection of the Chinese Academy of Science. ESCC cells were cultured in RPMI-1640 medium (Biological Industries, China) supplemented with 10% inactivated FBS (Biological Industries, China) and 1% penicillin/streptomycin. The cells were cytogenetically tested by STR- Promega and were authenticated (August, 2014 and July, 2017) [18, 19]. HEK293T cells (ATCC) were cultured in DMEM medium (Biological Industries, China). All cells were maintained at 37 °C in a humidified 5% CO_2_ incubator.

### Reagents and antibodies

Nuplazid was purchased from J&K Chemical (Beijing, China). jetPRIME® transfection reagent was purchased from Polyplus Transferion® SA. Protein A/G agarose beads was obtained from Santa Cruz. Antibodies to detect PAK4, p-PAK4 (Ser474) and MAPK1 were purchased from CST (Beverly, MA, USA). P-MAPK1 (Thr185/Tyr187) antibody was obtained from Thermo Scientific (Waltham, MA, USA). Antibody to detect Flag was purchased from HuaBio (Hangzhou, China). Ki67 antibody was obtained from Abcam (Cambridge, MA, USA).

### Cytotoxicity assay and cell proliferation assay

Cells (SHEE, KYSE150 and KYSE450; 8 × 10^3^/well) were seeded in 96-well plates with 0.1 ml of medium containing 10% FBS. After incubation for 16 h, 0.1 ml, a medium with different concentrations of Nuplazid was added. After incubation for another 24 h and 48 h for cytotoxicity assay and 24, 48, 72 and 96 h for cell proliferation assay, the plates were removed from the incubator. Hundred microlitres of 1 μg/ml DAPI was added to each well after fixing the cells with 4% paraformaldehyde for 30 min. After incubation at 37 °C for 20 min, the cells were counted by a high content imaging system (In Cell Analyzer 6000, GE Healthcare).

### Anchorage-independent cell growth

The soft-agar colony formation assay was used to determine the anchorage-independent cell growth. Three millilitres Eagle Basal Medium with 10% FBS and 0.6% agar was added to each well of a six-well plate and solidified at room temperature for 2 h. ESCC cells (8 × 10^3^ /well) were suspended in 1 ml Eagle’s Basal Medium (BME) with 10% FBS and 0.3% agar and subsequently plated over the solidified bottom layer. After incubation at 37 °C in 5% CO_2_ for 7 days, the colonies were photographed and counted using a high content imaging system (In Cell Analyzer 6000, GE Healthcare).

### Plate clone formation assay

Cells (200/well) were seeded in six-well plates with 2 ml of medium containing 10% FBS. After 16 h incubation, 2 ml of medium with different concentrations of Nuplazid was added to each well. After incubation for 7 days, the plates were removed from the incubator and the clones were stained with 0.5% crystal violet for 15 min after fixation with 4% paraformaldehyde for 30 min. Then the clones were photographed and counted.

### Cell sample preparation and Phosphoproteome analysis

KYSE150 (4.5 × 10^6^ cells) were seeded into a 15 cm dish and treated with 5 μM Nuplazid or DMSO for 24 h as control. For trypsin digestion, protein was extracted from cells using buffer with 8 M urea with 1% protease inhibitor cocktail. After reduction with 5 mM dithiothreitol at 56 °C for 30 min, the protein sample was alkylated with 11 mM iodoacetamide for 15 min RT in darkness. Then 100 mM TEAB was added to dilute samples to a urea concentration less than 2 M. Trypsin was added to the sample (according to mass ratio 1:50 trypsin-to-protein) for the first digestion overnight and 1:100 trypsin-to-protein mass ratio for a second 4 h digestion. After digestion with trypsin, peptide was desalted by Strata X C18 SPE column (Phenomenex) and vacuum-dried according to the manufacturer’s protocol for TMT kit/iTRAQ kit. After reconstitution in 0.5 M TEAB and processing by TMT kit/iTRAQ kit (Thermo, 90064), peptides were fractionated by high pH reverse-phase HPLC using Agilent 300Extend C18 column (5 μm particles, 4.6 mm ID, 250 mm length). The peptides were subjected to NSI source followed by tandem mass spectrometry (MS/MS) in Q ExactiveTM Plus (Thermo) coupled online to the UPLC.

### Data analysis

The resulting MS/MS data were processed using Maxquant search engine (v.1.5.2.8). The results were exported into Excel to facilitate database research. KEGG pathway research was conducted to analyse quantitative protein and phosphorylation sites. *P* < 0.05 was evaluated for statistical significance. FDR was set to <1%.

### Knockdown of PAK4 by shRNA

The shRNA against human PAK4 was inserted into the pLKO.1 vector. Human shPAK4 full hairpin sequence is #2 CGAGAATGTGGTG GAGATGTA, #5 CGACCAGCACGAGCAGAAGTT. HEK293T cells were transiently transfected with pLKO.1 or shPAK4 plus pMD2.G and psPAX2. The cells were placed in fresh medium after 4 h. Then the lentivirus enriched culture medium was collected at 24 and 48 h post-transfection and stored at −80 °C until required for use. The KYSE150 and KYSE450 cells were seeded and infected with lentivirus medium supplemented with polybrene (8 µg/mL; YESEN). After 48 h, the infected cells were selected in complete growth medium supplemented with 2 μg/mL of puromycin (Solarbio) for 24–48 h.

### Overexpressing of PAK4

KYSE410 was chosen to construct PAK4 overexpression cell line by screening the expression level of PAK4 in ESCC cell lines. KYSE410 cell (2 × 10^6^ cells) was infected using pLVX-IRES-puro-PAK4 full length plasmid and pLVX-IRES-puro vector as control, the plasmids were transfected into KYSE410 cells using jetPRIME® transfection reagent. After 48 h, KYSE410 cells were harvested to perform western blot, cell proliferation (1.5 × 10^3^ per well), plate clone formation (2 × 10^2^ per well) and soft-agar assay (8 × 10^3^ per well).

### Immunoprecipitation

KYSE150 and KYSE450 (2 × 10^6^ cells) were seeded into a 10 cm dish and treated with 5 μM nuplazid or DMSO for 24 h as control. Cell pellets were incubated with lysis buffer (50 mM Tris-HCl pH 8.0, 0.5% NP40, 150 mM NaCl) for 30 min at 4 °C. After quantification, appropriate cell lysates were incubated with 40 μL of protein A/G agarose beads and rotated for 2 h at 4 °C. Then collect cell lysate and add PAK4 antibody, 40 μL of protein A/G agarose beads was then added to each sample and rotated overnight at 4 °C. The beads were washed four times with lysis buffer, and the immune complexes were eluted at 95 °C for 5 min with 6×loading buffer. The immunoprecipitated complexes were then separated by SDS/PAGE and subjected to western blot analysis.

### Pull-down assay

Nuplazid-conjugated Sepharose 4 B beads or Sepharose 4 B beads were incubated with PAK4 protein or cell lysates (500 µg) in reaction buffer (2 mg/mL BSA, 50 mM Tris-HCl pH 7.4, 200 mM NaCl, 5 mM EDTA, 1 mM dithiothreitol, and 0.01% NP40) at 4 °C overnight. After the beads were washed three times by wash buffer (200 mM NaCl, 50 mM Tris pH 7.4, 5 mM EDTA, 0.01% NP40, and 1 mM dithiothreitol), the proteins were eluted from the beads through boiling in loading buffer at 95°C for 5 min. Protein binding was then assessed using Western blotting.

### Western blot analysis

Cells were seeded into a 15 cm dish at 4.5 × 10^6^ cells/well and treated with Nuplazid for 24 h. Total protein was extracted from cells using RIPA lysate buffer. Protein samples (30 μg, quantified by the BCA Protein Assay Kit) were boiled with loading buffer at 98 °C for 8 min and then were subjected to SDS–PAGE and transferred to a PVDF membrane (Immobilon®-P Membrane). After blocking with 5% non-fat milk for 1 h at room temperature, the membrane was incubated with primary antibody at 4 °C overnight. The next day, the membrane was incubated with the corresponding secondary antibody at room temperature for 2 h after being washed three times with TBST. Protein bands were visualised using the enhanced chemiluminescence (ECL, Meilunbio®).

### In vitro kinase assay

The activity of PAK4 was determined in a kinase reaction buffer with 200 μM ATP, in the presence of the kinase (100 ng of His-PAK4) and 200 ng of the substrate (His-MAPK1) at 30 °C for 30 min. The reaction was stopped by adding sample loading buffer and heating at 100 °C for 5 min. The kinase activity was visualised by detecting the indicated antibodies with -western blotting.

### Computational modeling of PAK4 with Nuplazid

For in silico docking of Nuplazid and PAK4, we used the Maestro 11.5 software program. To model the binding of Nuplazid with PAK4, the crystal structure of PAK4 (PDB number 2cdz) was downloaded from the PDB database (www.rcsb.org/pdb [PDB]). And water molecules in the structure were removed and hydrogen atoms were added to the protein prior to docking. The structure of Nuplazid was downloaded from PubChem database (https://pubchem.ncbi.nlm.nih.gov/) for the docking study. The PyMOL program was used to prepare the Nuplazid for the docking study.

### PDX mouse model

This study was approved under guidelines established by the Bioethics Committee of Zhengzhou University and followed guidelines set by the Institutional Animal Care and Use Committee (CUHCI2019002, CUHCI2021001, and CUHCI2021005). SCID-CB17 mice were purchased from Charles River and kept under specific pathogen-free conditions. ESCC tissues were obtained from the First Affiliated Hospital of Zhengzhou University, and written informed consent for the use of the tissue samples was provided by all patients. ESCC fragments of ~1–2 mm^3^ were seeded under the skin of the mice. Mice were randomly divided into three groups of eight animals each as follows: (1) untreated vehicle group; (2) 11 mg Nuplazid/kg of body weight and (3) 44 mg Nuplazid/kg body weight. Mice were given Nuplazid or vehicle (10% DMSO in 0.9% saline) by gavage every day. Tumour volume was calculated by the following formula: tumour volume (mm^3^) = (length × width^2^)/2. When the tumour volume reached ~1000 mm^3^, the mice were anesthetised, and the tumour weight was measured.

### Immunohistochemistry analysis

After fixation with 4% formaldehyde for at least 48 h, tumour tissues were embedded in paraffin blocks and subjected to immunohistochemistry (IHC). Serial 4 μm paraffin tissue sections were rehydrated using alcohol and TBST after being deparaffinised at 60 °C for 2 h. Next, the slides were boiled in sodium citrate buffer solution for 15 min. Afterwards, the tissue sections were treated with 3% H_2_O_2_ for 8 min. For IHC, tissues were hybridised with the Ki67 and p-MAPK1^T185/Y187^ primary antibody (1:50) at 4 °C overnight. Then the slides were incubated with HRP-IgG secondary antibody after being washed with TBST. Finally, the slides were stained with diaminobenzidine (DAB) for 2 min and then counterstained with hematoxylin. All sections were scanned using Tissue Faxes (TissueGnostics, version 4.2) and the Image Pro Plus software program (Media Cybernetics, Rockville, MD) was used to calculate positive cells.

### Statistical analysis

All quantitative results are expressed as mean values ± SD from three times replicates. SPSS 21.0 was applied to evaluate significant difference. One-way ANOVA or a non-parametric test was used for statistical analysis; *p* < 0.05 was considered statistically significant.

## Results

### Nuplazid inhibited the proliferation of ESCC cells

**Fig. 1:**
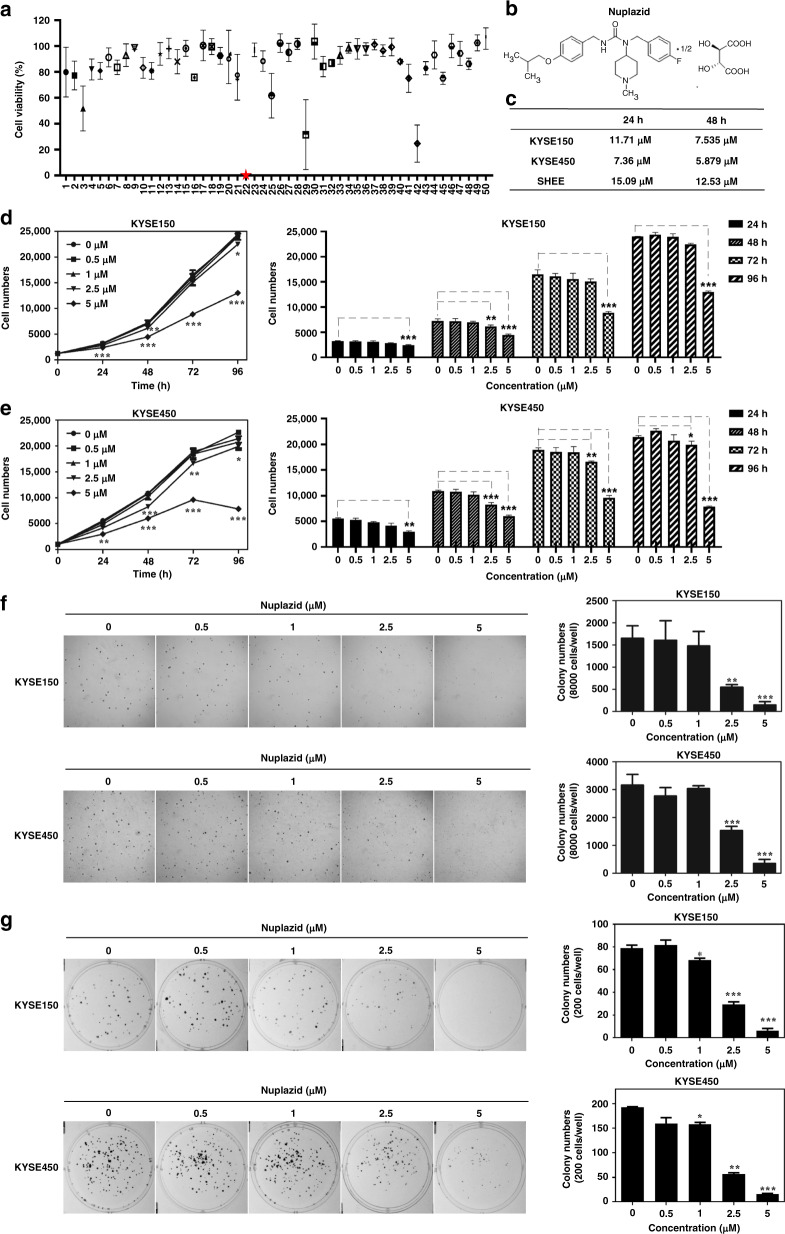
Nuplaid inhibits the proliferation of ESCC cells. **a** Nuplazid was screened from the drug library by cell cytotoxicity. **b** Chemical structure of Nuplazid. **c** IC50s of Nuplazid in KYSE150 cells, KYSE450 cells and SHEE cells. IC50s were calculated based on day 5 data of various doses of drug treatment. The KYSE150 cells (**d**) and KYSE450 cells (**e**) were treated with different doses of Nuplazid (0, 0.5, 1, 2.5 and 5 µM) and cell numbers were calculated at 0, 24, 48, 72 and 96 h by analysis at IN Cell Analyzer 6000. **f** KYSE150 cells and KYSE450 cells (8 × 10^3^/well) were exposed to different concentrations of Nuplazid (0, 0.5, 1, 2.5 and 5 µM) for 8 days. Colonies were counted for analysis by IN Cell Analyzer 6000 soft-agar program. **g** KYSE150 and KYSE450 cells (200/well) were treated with different concentrations of Nuplazid (0, 0.5, 1, 2.5 and 5 µM) and incubated for 8 days. Colonies were detected using the crystal violet stain assay. All data are shown as means ± S.D. The asterisks (*, **, ***) indicate a significant decrease (*p* < 0.05, *p* < 0.01, *p* < 0.001, respectively).

To select a drug that can effectively suppress ESCC proliferation, we screened an FDA-approved drug library with a toxicity assay. We found Nuplazid (compound number 22) exhibited toxic effects on KYSE450 cells (Fig. 1a). The specific information of the compound is in Table 1 of the Supplementary data. Nuplazid is a 5-HT 2 A receptor inverse agonist (Fig. 1b) that is mainly used to treat Parkinson’s disease. The IC50 of Nuplazid on ESCC cells and immortalised epithelial cells was evaluated by a cell cytotoxicity assay. The results indicated that Nuplazid exhibited more toxic effects on KYSE150 and KYSE450 cells compared to SHEE cells (Fig. 1c and Fig. S1). To determine the inhibitory effect of Nuplazid on ESCC, KYSE150 and KYSE450 cells were treated with Nuplazid (0, 0.5, 1, 2.5 and 5 µM). The results indicated that Nuplazid effectively suppressed the growth of ESCC cells in a dose-dependent manner (Fig. 1d, e). We next investigated whether Nuplazid could inhibit anchorage-independent growth of ESCC cells. The soft-agar assay showed that Nuplazid could significantly block the anchorage-independent growth of KYSE150 and KYSE450 cells in a dose-dependent manner (Fig. 1f). In addition, the growth inhibitory effect of Nuplazid on ESCC was evaluated. The results of the plate clone formation assay also indicated that clone formation in KYSE150 and KYSE450 cells was inhibited after Nuplazid treatment (Fig. 1g). Together, these results indicate that Nuplazid inhibits ESCC cell growth in vitro.

**Table 1:** The table showed the survival rate of KYSE450 cells which were treated with 50 µM different compounds for 48 h (*n* = 3).

	Compound	Cell viability	Error amount
1	Oxytetracycline	79.88741	19.22323
2	Furaltadone hydrochloride	77.24526	11.11366
3	Nitrofurazone	51.82093	17.36424
4	Chloroxylenol	82.00295	8.014399
5	Benzocaine	80.89498	6.461878
6	Chlorobutanol	91.27302	7.388945
7	Ofloxacin	83.53816	5.590931
8	Topiramate	92.96267	8.695055
9	Betahistine mesylate	98.62139	1.031306
10	Adrenosterone	83.23309	8.027643
11	Spectinomycin dihydrochloride	73.83655	9.928254
12	Dihydrostreptomycin sulfate	93.95017	8.7414
13	Guaifenesin	98.01176	7.893334
14	Adenosine 5′-monophosphate	87.95408	9.379032
15	Geniposide	98.22132	6.085869
16	Flumethasone	75.76876	2.51898
17	Sparteine sulfate pentahydrate	100.395	11.87379
18	Kanamycin sulfate	99.56081	6.104918
19	Alodan	92.51785	6.457572
20	Terconazole	91.62695	20.55539
21	Phenacetin	75.90553	17.62029

Number	Compound	Cell viability	Error amount
22	**Pimavanserin Tartrate**	**0.477261**	**0.66110811**
23	Pyridoxine hydrochloride	97.07729	5.264602
24	Salicylic acid	88.3694	7.935546
25	Magnolol	61.72762	17.17745
26	Gastrodin	102.2996	7.196228
27	Piperine	95.13027	7.026235
28	Chrysophanic Acid	101.6242	4.183081
29	Terandrine	31.56692	26.94679
30	Ligustrazine hydrochloride	103.4953	13.47551
31	Proanthocyanidins	84.17642	7.867275
32	Glycyrrhetinic acid(Enoxolone)	86.90683	2.867116
33	Chondroitine sulfate	92.9459	6.847844
34	Scoparone	98.63513	4.068273
35	Mepenzolate bromide	97.80974	7.847292
36	Benzthiazide	97.71037	4.752846
37	Limonin	101.2372	4.037471
38	Imperatorin	96.49294	4.462271
39	Puerarin(Kakonein)	99.2533	6.754354
40	Baicalein	88.01013	1.756159
41	Sulfasalazine	75.18233	10.88988
42	Chlorquinaldol	24.70887	14.34594

Number	Compound	Cell viability	Error amount
43	Nalidixic acid	82.85737	5.105331
44	Cefotaxime sodium salt	93.12401	10.8226
45	Tetracycline hydrochloride	75.11068	4.611462
46	Sulfamerazine	100.0874	9.097454
47	Sulfaquinoxaline sodium	94.32887	9.286951
48	Trimebutine	86.24522	4.442833
49	Ambroxol	102.4955	6.136034
50	Sulfabenzamide	105.8144	8.254695

### Nuplazid affects PAK4/MAPK1 signaling

**Fig. 2:**
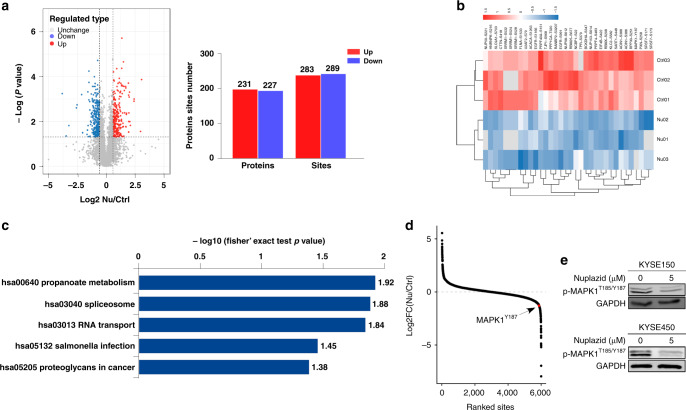
PAK4 kinase activity changes of KYSE150 cells after Nuplazid treatment. **a** Volcano plot shows that 572 unique phosphosites changed significantly (*t*-test *P*-value < 0.05, FDR < 0.01 upon 24 h treatment with 5 µM Nuplazid). Blue dots represent downregulated proteins, and red dots represent upregulated proteins, with 283 sites up and 289 sites down. **b** These phosphoproteins which were downregulated are mapped to Heat map upon Nuplazid treatment. **c** KEGG pathways that are significantly downregulated in phosphorylation are shown according to *P*-value. **d** Averaged quantitative phosphosites from the phosphoproteomic, the locations of MAPK1^Y187^ were ranked according to their Log2 FC between the DMSO and Nuplazid-treated cells. **e** Effect of Nuplazid treatment for 24 h on p-MAPK1^T185/Y187^ in KYSE150 and KYSE450 cells.

To investigate the anticancer mechanism of Nuplazid in ESCC cells, KYSE150 cells were treated with 5 μM Nuplazid or DMSO for 24 h as a control. Subsequently, we analysed proteomic and phosphorylation changes after Nuplazid treatment by performing mass spectrometry. The quality control report indicated that this test was in line with the standards (Fig. S2A and B). According to proteomic and phosphoproteomic analysis, 5165 proteins were quantified from 6449 proteins, and 5988 phosphorylation sites were quantified from 8852 sites. To evaluate statistical significance, strict criteria (*t-*test *P*-value < 0.05, FDR < 0.01) were applied to three biological replicates. Among all the quantified phosphosites, we discovered that 572 phosphosites were changed by 283 sites up and 289 sites down according to *P*-value < 0.05 (fold change > 1.5 or fold change < 0.67) (Fig. 2a). A series of phosphosites were differentially expressed in KYSE150 cells after Nuplazid treatment (Fig. 2b). The quantified phosphorylation sites were mapped to the KEGG signaling pathway, and the top five signaling pathways are shown in a KEGG pathway enrichment map (Fig. 2c). Based on the dataset, all changed phosphorylation sites are determined and the phosphorylation site of MAPK1 at 187 was a visible downregulated site (Figs. 2d and S2C). Western blot analysis revealed that the level of p-MAPK^Y187^ was decreased in KYSE150 and KYSE450 cells treated with Nuplazid for 24 h (Fig. 2e). To identify the upstream kinases that regulate these proteins, the upstream kinase was predicted based on changes in protein phosphorylation sites, which showed that the kinase activity of PAK4 may be regulated by Nuplazid (Fig. S3). Meanwhile, Swiss Target also predicted PAK4 may be the target protein of Nuplazid (Fig. S3). We found that PAK4 and MAPK1 were positively correlated by TCGA database analysis (Fig. S3). Therefore, we hypothesised that Nuplazid may target PAK4 and regulate its downstream signaling pathway.

### Nuplazid could bind to PAK4

**Fig. 3:**
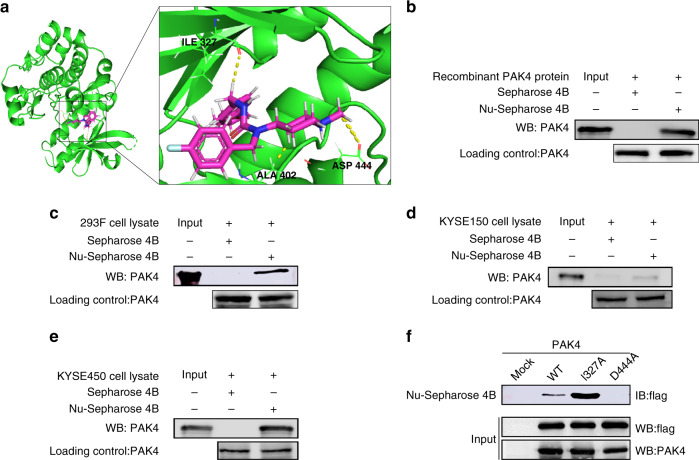
Nuplazid directly binds to PAK4. **a** Modeling of Nuplazid binding with PAK4. The recombinant protein or cell lysate was incubated with Nuplazid‐conjugated Sepharose 4B beads or with Sepharose 4B beads alone. Nuplazid directly binds to PAK4 in recombinant proteins (**b**) or 293 F cell lysates which overexpressed PAK4 (**c**) or KYSE150 lysate (**d**) or KYSE450 lysate (**e**) or cells (**f**) ectopically expressing PAK4 (WT, mutant I327A, or D444A). Proteins were pulled down and then analysed by western blotting using antibodies to detect PAK4. For **b**–**f**, similar results were obtained from independent experiments.

To better understand how Nuplazid binds with PAK4, we created a computational docking model through Maestro 11.5. The docking result indicated that Nuplazid formed hydrogen bonds with PAK4 (Fig. 3a). Then in vitro pull-down assay was performed to confirm the computational docking results of Nuplazid with PAK4, we used Nuplazid-conjugated Sepharose 4 B beads (or Sepharose 4 B beads only as a control) and a recombinant PAK4 protein (Fig. 3b), HEK293F cell lysates that overexpressed PAK4 (Fig. 3c), KYSE150 lysate (Fig. 3d), or KYSE450 lysate (Fig. 3e). These results indicated Nuplazid can bind to PAK4 in vitro and ex vivo. The previous docking result indicated that Nuplazid might bind with PAK4 at ATP-binding sites ILE327, ALA402 and ASP444. Therefore, mutant PAK4 (I327A, D444A) was constructed and ectopically expressed in HEK293 F cells. Then pull-down assays using Nuplazid-conjugated Sepharose 4 B beads and HEK293F cell lysates that overexpressed PAK4 wild type protein or PAK4 mutant protein revealed that D444 A of PAK4 had the greatest reduction binding affinity with Nuplazid (Fig. 3f), suggesting that the ASP444 site is important for the binding of Nuplazid with PAK4. Our results indicated that Nuplazid directly binds to PAK4.

### Nuplazid inhibited the kinase activity of PAK4

**Fig. 4:**
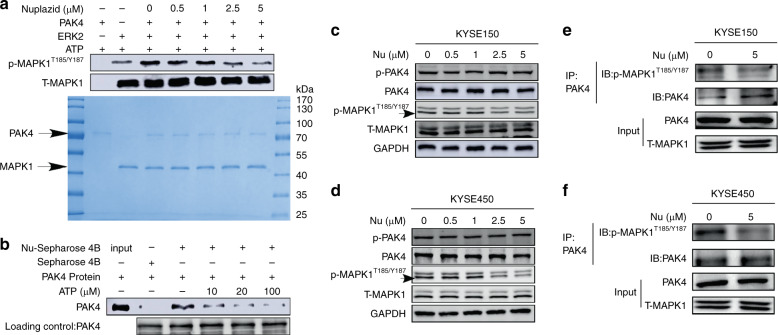
Nuplazid inhibits the kinase activity of PAK4. **a** PAK4 kinase activity was assessed by an in vitro kinase assay using active PAK4 and inactive MAPK1 proteins. The effect of Nuplazid was determined by western blot analysis using a p-MAPK1^T185/Y187^ antibody. **b** Nuplazid binds with PAK4 in an ATP-competitive manner. Active PAK4 was incubated with ATP at different concentrations (10, 20 or 100 µM) and 100 µl of Nuplazid–sepharose 4B or sepharose 4B (as a negative control) in reaction buffer. The level of p-PAK4, PAK4, p-MAPK1 and T-MAPK1 in KYSE150 cells (**c**) and KYSE450 cells (**d**) with different concentration of Nuplazid (0, 0.5, 1, 2.5 and 5 μM) treatment for 24 h was determined by western blotting. The level of p-MAPK1^T185/Y187^ was affected by PAK4 in KYSE150 cells (**e**) and KYSE450 cells (**f**) which treated with Nuplazid. MAPK1 was immunoprecipitated by PAK4 and MAPK1 was detected by p-MAPK1^T185/Y187^.

To identify whether Nuplazid could regulate the kinase activity of PAK4, we used a recombinant active PAK4 protein and inactive MAPK1 protein to perform an in vitro kinase assay. The results verified that Nuplazid could suppress the phosphorylation of MAPK1 (Fig. 4a). Because Nuplazid inhibited PAK4 kinase activity and could bind with PAK4 at ATP-binding sites ASP444, we performed pull-down assays with ATP to investigate how Nuplazid interacts directly with PAK4 kinase. The binding of Nuplazid to PAK4 decreased in the presence of ATP (Fig. 4b), indicating that Nuplazid suppresses the kinase activity of PAK4 by an ATP-competitive manner. We next determined whether the PAK4 downstream signaling in ESCC cells could be affected by Nuplazid. After treatment with Nuplazid for 24 h, the proteins in KYSE150 and KYSE450 cells were extracted and PAK4 signaling pathway molecules were detected by Western blotting. Results indicated Nuplazid inhibited MAPK1 phosphorylation in a dose-dependent manner, whereas PAK4 and phosphorylated PAK4 remained unchanged (Fig. 4c, d). To further determine whether Nuplazid could affect PAK4 interact with MAPK1, KYSE150 cells and KYSE450 cells treated with Nuplazid. PAK4 was used to immunoprecipitate MAPK1 and p-MAPK1^T185/Y187^ was used to detect p-MAPK1^T185/Y187^ by Western blot analysis. The results indicated that PAK4 can combine with MAPK1 and Nuplazid could decreased the level of p-MAPK1^T185/Y187^ through affected the kinase activity of PAK4 (Fig. 4e, f).

### Nuplazid suppressed ESCC growth through PAK4

**Fig. 5:**
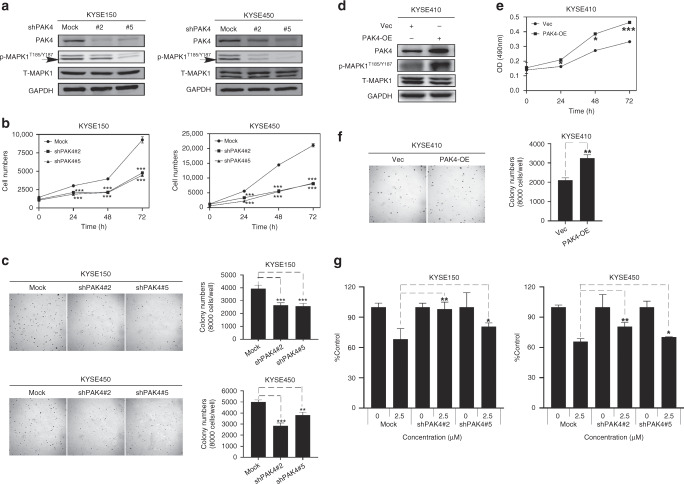
Nuplazid inhibits ESCC growth through PAK4. **a** The knockdown efficiency of PAK4 by shRNA (#2, #5) in KYSE150 and KYSE450 cells was evaluated by Immunoblotting. And the level of the PAK4 downstream signal MAPK in the shPAK4 cells was detected by WB. **b** Cell numbers of KYSE150 and KYSE450 cells with transfected shPAK4 at 0, 24, 48 and 72 h. **c** Colonies were counted for KYSE150 and KYSE450 cells with transfected with shPAK4 after 7 days. **d** The overexpression efficiency of PAK4 in KYSE410 cells was evaluated by WB and the level of the PAK4 downstream signal MAPK1 in the KYSE410 cells with transfected PAK4 was detected by WB. **e** Cell numbers of KYSE410 cells with transfected PAK4 at 0, 24, 48 and 72 h. **f** Colonies were counted for KYSE410 cells with transfected PAK4 after 7 days. **g** The KYSE150 cells and KYSE450 cells were treated with 2.5 μM Nuplazid for 96 h, and the inhibition rate was calculated. All data are shown as means ± S.D. The asterisks (*, **, ***) indicate a significant decrease (*p* < 0.05, *p* < 0.01, *p* < 0.001, respectively).

To investigate whether PAK4 knockdown might mediate the growth of ESCC, we established multiple PAK4 knockdown cell lines. Western blotting results showed that the expression of PAK4 in cells transfected with two individual PAK4 shRNA was significantly decreased compared with control cells (Fig. 5a). And the level of p-MAPK1^T185/Y187^ was decreased in PAK4 knockdown cells (Fig. 5a). Then we used cell proliferation and anchorage-independent cell growth assays to assess whether the knockdown of PAK4 inhibits the growth of ESCC. Cells with PAK4 knockdown exhibited slower growth and colony formation compared to control sh-mock cells (Fig. 5b, c). To further provide PAK4 plays an active role in ESCC, we assessed whether overexpression of PAK4 could promote cell proliferation in ESCC cells. First, we detected the expression levels of PAK4 in different ESCC cell lines, and the results showed that PAK4 was lowly expressed in KYSE410 cells (Fig. S4). Then we established PAK4 overexpressing KYSE410 cells. The Western blot result showed that the expression of PAK4 in KYSE410 cells was significantly higher than control cells that expressed the empty PLVX-IRES-puro vector (Fig. 5d) and the level of p-MAPK1^T185/Y187^ was increased in PAK4 overexpressing cells (Fig. 5d). As expected, overexpression of PAK4 significantly promoted the cell growth (Fig. 5e) and colony formation (Fig. 5f) compared to control cells. Taken together, these data demonstrated that the PAK4 play an important role in ESCC cells.

To verify whether Nuplazid affects the proliferation of esophageal cancer cells through PAK4, shPAK4-KYSE150, shPAK4-KYSE450 cells and their control sh-mock cells were treated with 2.5 μM Nuplazid. The results indicated the growth of cells with PAK4 knockdown was less inhibited compared to control sh-mock cells (Fig. 5g). In summary, Nuplazid inhibits ESCC growth through PAK4.

### Nuplazid suppressed ESCC PDX tumour growth in vivo

**Fig. 6:**
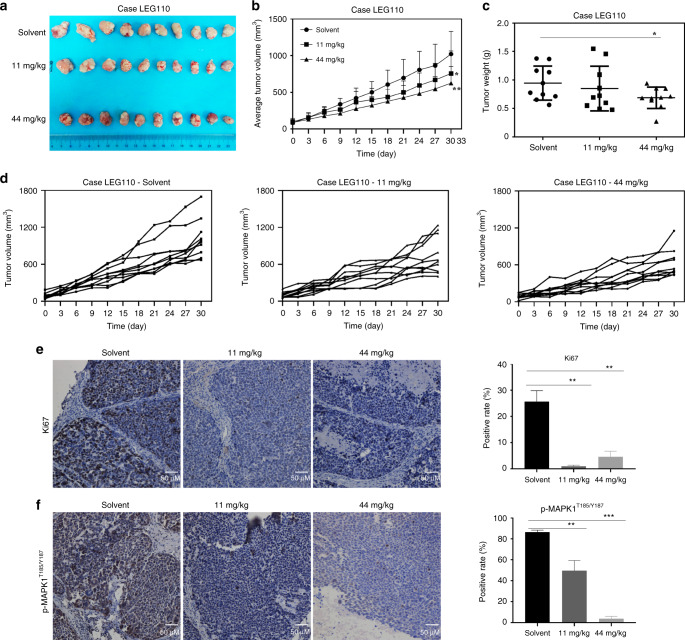
Nuplazid inhibits the growth of esophageal cancer patient-derived xenograft tumours in vivo. **a** The photograph showed tumour tissues from PDX mice treated with solvent or Nuplazid (11 mg/kg or 44 mg/kg). **b** Tumour volumes were measured every 3 days. **c** After sacrificing, isolated tumours were weighted. **d** Data recorded tumour size of individual mice. Immunohistochemistry analysed the level of ki67 (**e**) and p-MAPK1^T185/Y187^ (**f**) in tumour tissues from treated or untreated groups of mice. All data are shown as means ± S.D. The asterisks (*, **, ***) indicate a significant decrease (*p* < 0.05, *p* < 0.01, *p* < 0.001, respectively).

To examine the anti-tumour activity of Nuplazid against the growth of ESCC tissues in vivo, ESCC PDXs models were established in mice. To determine the dose of Nuplazid for the in vivo experiment, we performed a potential toxicity profile of Nuplazid. Based on the daily oral dose in humans, mice were administered Nuplazid at 11 mg/kg or 44 mg/kg every day by gavage for 2 weeks. The results indicated that there was no body weight change in mice between the Nuplazid-treated group (11 mg/kg or 44 mg/kg) and the vehicle-treated group (Fig. S5). Therefore, mice were administered Nuplazid at either 11 mg/kg or 44 mg/kg daily by gavage over a period of 30 days. The results indicated that the Nuplazid treatment significantly suppressed tumour growth compared with the vehicle-treated group (Fig. 6a, b). Moreover, tumour weight was significantly decreased in the Nuplazid 44 mg/kg treatment group compared with the vehicle-treated group (Fig. 6c). Meanwhile, the results indicated that although the tumour in the vehicle-treated group grew rapidly, Nuplazid reduced the growth of most tumours (Fig. 6d). Next the effect of Nuplazid on the expression of Ki67 was investigated by IHC, and the results indicated that the Nuplazid treatment significantly inhibited the expression of Ki67 in tissues compared with the vehicle-treated group (Fig. 6e). The effect of Nuplazid on the PAK4 signaling pathway in tumour tissues was examined, and the results indicated that the Nuplazid treatment strongly suppressed the phosphorylation of MAPK1 in LEG110 tumours (Fig. 6f).

## Discussion

Esophageal cancer is one of the most common malignant tumours, with an estimated 572,000 new cases and 509,000 deaths annually [20]. Despite recent advances in treatments, patient prognosis remains poor [21–23]. Therefore, it is urgent to identify effective agents for ESCC prevention or recurrence prevention [24].

Drug repurposing has the potential to overcome several challenges associated with de novo drug discovery and guarantees quick clinical trials due to the already-established pharmacokinetics, tolerability, safety, and toxicity profile of the drug. Therefore, screening the FDA-approved drug library to select low-toxicity and high-efficiency drugs for cancer prevention or recurrence prevention is extremely attractive. Through this strategy we discovered that Nuplazid was cytotoxic against ESCC cells and that it suppressed ESCC proliferation and clone formation in vitro. Nuplazid is an effective 5-HT 2 A receptor inverse agonist that is clinically used to treat Parkinson’s disease psychosis [25]. Recently, a study reported that Nuplazid exerts anticancer effects in pancreatic cancer cells [26]. However, the molecular targets and the inhibitory mechanism of Nuplazid remain to be elucidated.

Here, by analyzing phosphoproteome data after Nuplazid treatment and predicting the upstream kinase of the protein mapped to the signal pathway, we found that the activity of PAK4 changed significantly. Moreover, Swiss target analysis indicated that Nuplazid binds with PAK4. Interestingly, the binding assays and in vitro kinase assay provided strong evidence that Nuplazid targeted PAK4 and inhibited its activity. Interestingly, we found that MAPK1 can be phosphorylated by PAK4 at Y187, which was proved by in vitro kinase assay. MAPK1^Y187^ levels can be suppressed by Nuplazid treatment. All together, the PAK4 signaling pathway in ESCC cells was inhibited after Nuplazid treatment.

Accumulated evidence supports a critical role for abnormal PAK4 expression in oncogenesis, and amplification or activation of PAK4 has been detected in numerous cancers [27–29], including pancreatic, breast, and ovarian cancers [30]. Recent studies have indicated that PAK4 is associated with the risk of ESCC, and TCGA database analysis indicated that PAK4 expression in ESCC tissues was higher than in normal tissues [31]. We found that knockdown of PAK4 significantly suppressed the growth of ESCC cells, and knockdown of PAK4 led to ESCC cell resistance to Nuplazid. These multiple pieces of evidence indicated that PAK4 is a promising target of ESCC.

Fluorouracil, capecitabine, oxaliplatin and paclitaxel are often used in combination as chemotherapeutic agents for ESCC [32–36]. However, these drugs have side effects on the patient’s physical and mental state, and the gradual development of drug resistance is also a challenge for clinical treatment [37]. Recently, studies have reported that some PAK4 molecular inhibitors, LCH-7749944 [15], FRAX1036 [38], and GNE 2861 [39], showed inhibitory effects in melanoma cells, colon cancer cells, breast cancer cells, and gastric cancer cells. However, these PAK4 inhibitors are still in preclinical research. Thus, a new PAK4 inhibitor that is not inferior in efficacy but that is favourable in toxicity is urgently needed. Nuplazid is a clinical drug already approved by the FDA with established safety data. Importantly, in the in vivo experiment, we proved that Nuplazid significantly suppressed tumour growth in mice PDXs at its clinical dose, which paved its way for further clinical trials.

In conclusion, our findings support the idea that Nuplazid is a potent PAK4 inhibitor, and could be used in ESCC chemoprevention.

## Supplementary information

supplementary material

## Data Availability

The data supporting the findings of this study can be found in the article, Supplementary Information or available from the corresponding author upon reasonable request.
