# Supplementary material for: Nuplazid suppresses esophageal squamous cell carcinoma growth in vitro and in vivo by targeting PAK4
Source: Br J Cancer. 2021 Dec 15;126(7):1037–46. doi: 10.1038/s41416-021-01651-z (PMC8980085; doi:10.1038/s41416-021-01651-z)
Supplement: Supplementary file 1 — supplementary material [file 41416_2021_1651_MOESM1_ESM.docx]

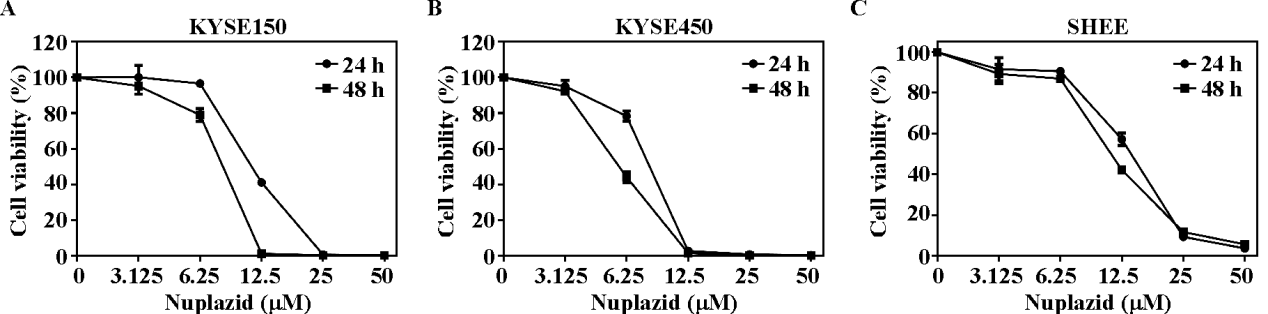


Figure. S1. The KYSE150 cells (A), KYSE450 (B) cells and SHEE cells (C) were treated with different doses of Nuplazid (0, 3.125, 6.25, 12.5, 25 and 50 µM) for 24 and 48 h, cell numbers were calculated a by analysis at IN Cell Analyzer 6000 and cell viability were caculated.


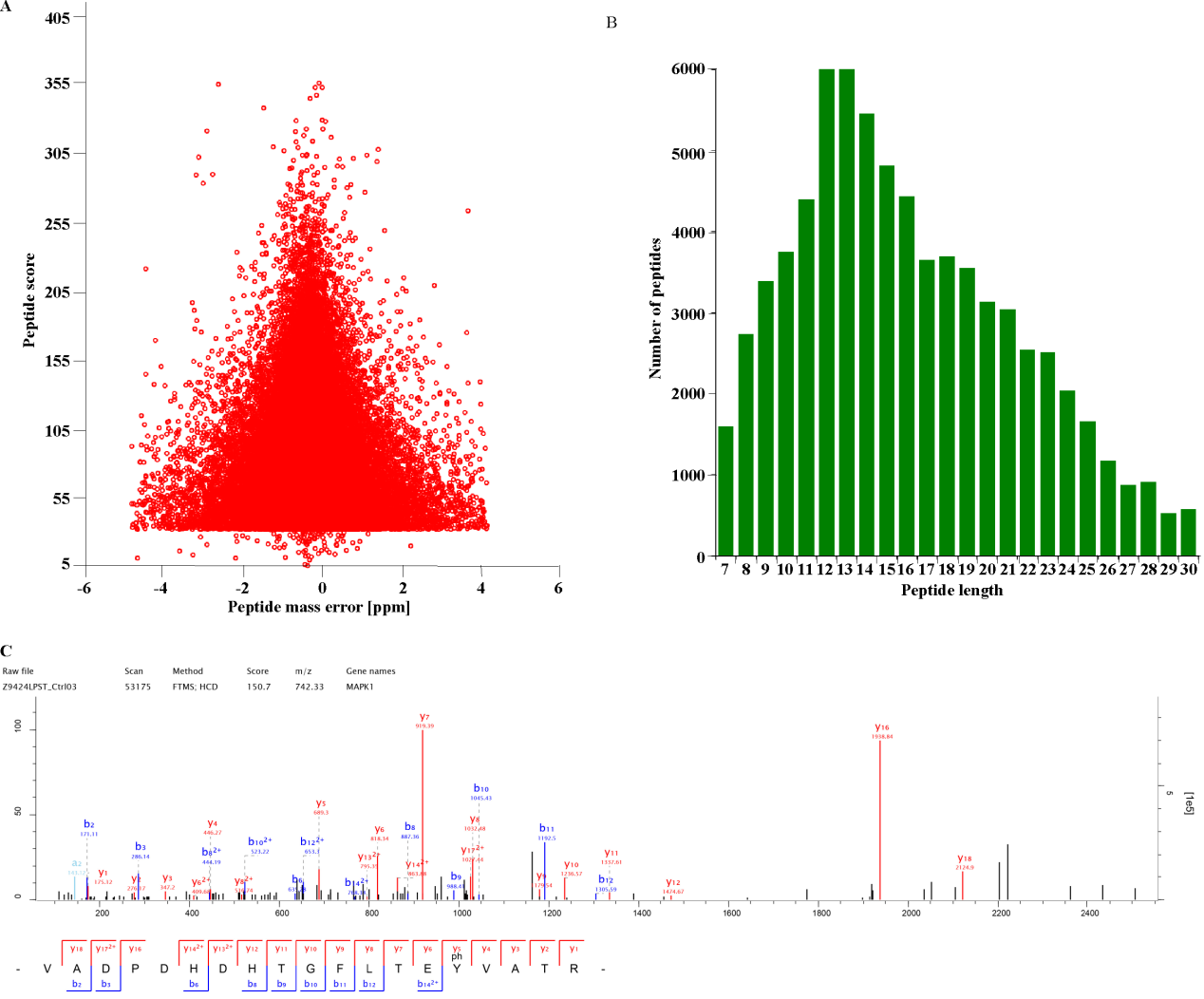


Figure. S2. (A) Identificated peptide mass offset distribution map. (B) Identified peptide length distribution map. (C) Identification of phosphorylation site of MAPK1 by HPLC- MS/MS spectrometry.


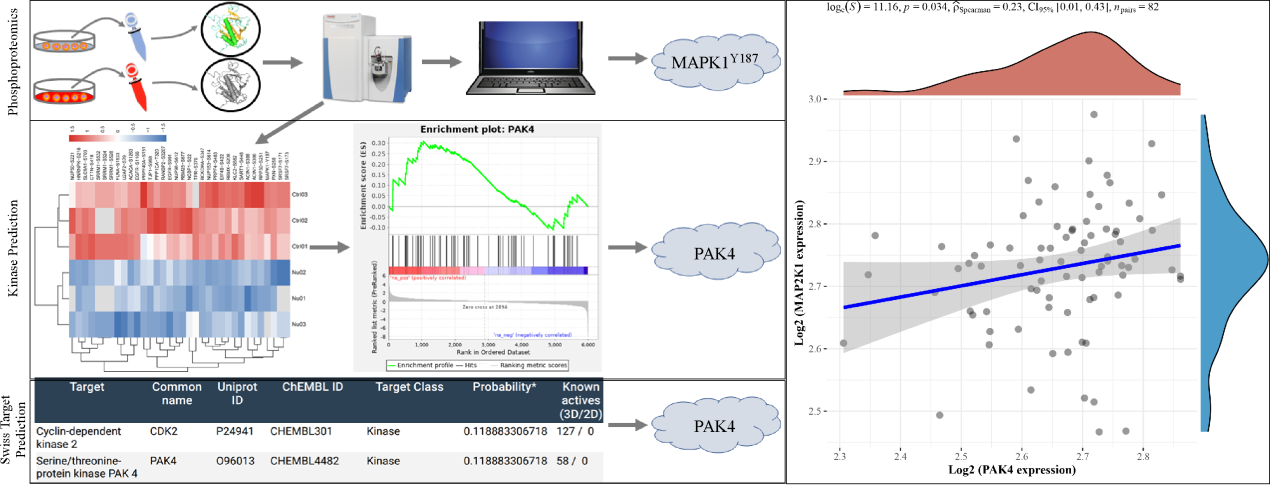


Figure. S3. Schematic diagram of the quantitative phosphoproteomic analysis process and MAPK1^Y187^ is affected by Nuplazd treatment through phosphorylation analysis. The upstream kinases regulating these phosphosites were predicted through GSEA. And the kinase activity of PAK4 is changed. The target of Nuplaizd is predicted by Swiss target. The correlation between PAK4 and MAPK1 was analyzed through TCGA database.


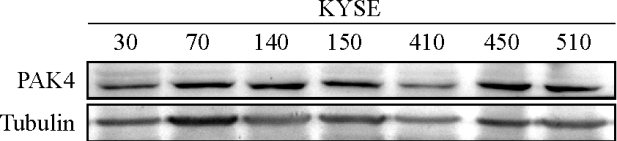


Figure. S4.The protein levels of PAK4 in different ESCC cell lines.


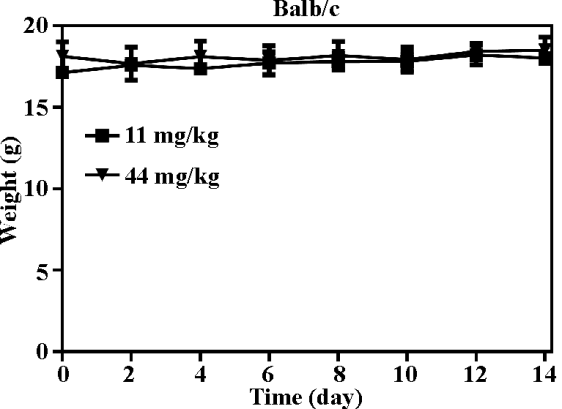


Figure S5. The body weight of mice was measured every 2 days.
